# Adiponectin is Associated with Impaired Fasting Glucose in the Non-Diabetic Population

**DOI:** 10.4178/epih/e2011007

**Published:** 2011-08-20

**Authors:** Sang Yeun Kim, Sun Ju Lee, Hyoun Kyoung Park, Ji Eun Yun, Myoungsook Lee, Jidong Sung, Sun Ha Jee

**Affiliations:** 1 Institute for Health Promotion, Graduate School of Public Health, Yonsei University, Seoul, Korea.; 2 Department of Epidemiology and Health Promotion, Graduate School of Public Health, Yonsei University, Seoul, Korea.; 3 Department of Food and Nutrition and Research Institute of Obesity Sciences, Sungshin Women's University, Seoul, Korea.; 4 Division of Cardiology, Department of Internal Medicine, Center for Health Promotion, Samsung Medical Center, Sungkyunkwan University School of Medicine, Seoul, Korea.

**Keywords:** Adiponectin, Diabetes, Impaired fasting glucose, Insulin resistance

## Abstract

**OBJECTIVES:**

Adiponectin is strongly associated with diabetes in the Western population. However, whether adiponectin is independently associated with impaired fasting glucose (IFG) in the non-obese population is unknown.

**METHODS:**

The serum adiponectin, insulin resistance (IR), and waist circumference (WC) of 27,549 healthy Koreans were measured. Individuals were then classified into tertile groups by gender. IFG was defined as a fasting serum glucose of 100-125 mg/dL without diabetes. IR was calculated using the homeostasis model assessment of insulin resistance (HOMA-IR). The association of adiponectin and IFG was determined using logistic regression analysis.

**RESULTS:**

WC and adiponectin were associated with IFG in both men and women. However, the association of WC with IFG was attenuated in both men and women after adjustment for the HOMA-IR. Adiponectin was still associated with IFG after adjustment for and stratification by HOMA-IR in men and women. Strong combined associations of IR and adiponectin with IFG were observed in men and women. Multivariate adjusted odds ratios (ORs) (95% confidence interval [CI]) among those in the highest tertile of IR and the lowest tertile of adiponectin were 9.8 (7.96 to 12.07) for men and 24.1 (13.86 to 41.94) for women.

**CONCLUSION:**

These results suggest that adiponectin is strongly associated with IFG, and point to adiponectin as an additional diagnostic biomarker of IFG in the non-diabetic population.

## INTRODUCTION

Diabetes is becoming a growing concern and worldwide health problem. According to World Health Organization (WHO) estimates, more than 220 million people worldwide have diabetes and an estimated 1.1 million people died from diabetes in 2005 [1]. Considering this epidemic, it is critical to understand what causes diabetes and to explore ways to effectively prevent it. Obesity and insulin resistance (IR) have been recognized as major risk factors for disease development. As a result, in order to predict diabetes, body mass index (BMI) and waist circumference (WC) have been used as obesity indicators. Obesity is defined as BMI of ≥25 kg/m^2^ and WC of ≥90 cm for men and ≥80 cm for women [2].

In conjunction with economic growth, the Korean population has recently experienced a rapid increase in obesity due to insufficient physical exercise, fast-food diets, and sedentary lifestyles. According to the Korean National Health and Nutrition Examination Survey [3] the mean BMI was 24.0 kg/m^2^ among men and 23.1 kg/m^2^ among women, while mean WC was 84.1 and 78.3 cm for men and women, respectively. Thus, more than half of the Korean population is considered non-obese according to WHO criteria. However, it is notable that some people still suffer from diabetes or impaired fasting glucose (IFG) even though their BMI and/or WC are categorized as normal.

According to a study that included 595 Asian individuals, of 297 subjects with impaired glucose tolerance (IGT) at baseline, 35.1% developed diabetes after eight years [4]. In another study, cumulative incidences of diabetes in subjects with IFG at baseline were 16.6% for men and 6.5% for women after four years [5]. To identify those at high risk for pre-diabetes several conditions are considered, including family history of diabetes, excess body weight (high BMI), abdominal adiposity (high WC) [6,7], high alcohol intake, and low physical activity [8]. Among these factors, BMI and WC have been reported as important predictors of IFG [9,10].

In contrast to Caucasians, the majority of Koreans with type 2 diabetes fall into the non-obese category [11,12] which may result in missed opportunities to detect those at a high risk of pre-diabetes, as the prevalence of pre-diabetes increases among populations with low WC and/or low BMI. Therefore, it is necessary to explore new biomarkers or indicators other than BMI or WC, which may help predict the onset of diabetes.

Recently, adiponectin level was introduced as a new indicator of obesity. In fact, several studies have reported that adiponectin was associated with diabetes independent of WC and BMI, introducing it as a useful marker for metabolic syndrome in type 2 diabetes in a Korean population [13,14]. Another robust finding identified a positive correlation between adiponectin and insulin sensitivity by hyperinsulinemic clamp [15,16] and the homeostasis model assessment of insulin resistance (HOMA-IR) [17]. It has also been reported that adiponectin is closely related to insulin sensitivity, independent of fat mass [16]; however, the importance of adiponectin and IR in non-diabetic and non-obese subjects remains to be elucidated.

The purpose of this study was to investigate the association between adiponectin, IR, and IFG among a non-diabetic population in Korea and to explore the possibility of using adiponectin as an additional biomarker to detect the pre-diabetic condition.

## MATERIALS AND METHODS

### Study subjects

The initial study population consisted of 32,248 subjects who participated in the Korean Metabolic Syndrome Research Initiatives (KMSRI) and volunteered to have routine health examinations at five health promotion centers in university hospitals (Severance, Ewha, Seoul National, Korea, and CHA) and one private health examination center (Korea Medical Institute) from January 2006 to December 2008. After excluding subjects with missing information related to smoking and alcohol intake and those with diabetes, data from 27,549 subjects were analyzed. The Institutional Review Board of Human Research of Yonsei University approved the study and written informed consent was obtained from all subjects.

### Data collection

Each participant was interviewed using a structured questionnaire to collect history regarding cigarette smoking (never smoked, ex-smoker, current smoker) and alcohol consumption (non-drinker or drinker of any amount of alcohol), as well as other demographic characteristics such as age, gender, and family history of diabetes. Participants were also asked if they did exercise (exercise, non-exercise). WC was measured midway between the lower rib and the iliac crest. Weight and height were measured while the participants were wearing light clothing. BMI was calculated as weight (kg) divided by the square of height (m^2^). The blood pressure was measured while in the seated position by a registered nurse or blood pressure technician using a standard mercury sphygmomanometer or automatic manometer. Both systolic and diastolic blood pressures were measured after a 15 minutes rest.

### Measurement of biomarkers

Serum was separated from peripheral venous blood samples obtained from each participant after 12 hr of fasting and stored at -70℃ prior to clinical chemistry assay. Metabolic syndrome biomarkers such as fasting blood glucose (FBG), total cholesterol (TC), triglycerides (TG), and high density lipoprotein cholesterol (HDL-C) were measured using a Hitachi-7600 analyzer (Hitachi Ltd., Tokyo, Japan). Adiponectin levels were measured in a central laboratory of KMSRI using an enzyme-linked immunosorbent assay (ELISA) (Mesdia Co., Ltd., Seoul, Korea). The intra- and inter-assay variances for adiponectin were 6.3% to 7.4% and 4.5% to 8.6%, respectively [18]. Data quality control was conducted in accordance with the procedures of the Korean Association of Laboratory Quality Control. IR was calculated using the HOMA-IR. HOMA indices were calculated as follows: HOMA=fasting insulin (µLU/mL)×fasting glucose (mmol/L)/22.5.

### Statistical analysis

Age-adjusted prevalence rates for IFG were calculated by direct method to the age distribution of the 2007 Korean adult population. Men and women were analyzed separately. The study samples were divided into tertile groups of WC (<81.0, 81.0-86.9, ≥87 cm for men; <70, 70-76.9, ≥77 cm for women), BMI (<23.1, 23.1-25.3, ≥25.4 kg/m^2^ for men; <20.9, 20.9-23.2, ≥23.3 kg/m^2^ for women), adiponectin (<4.7, 4.7-7.4, ≥7.4 µg/mL for men; <7.4, 7.4-11.9, ≥12 µg/mL for women), and HOMA-IR (<0.73, 0.73-1.22, ≥1.22 for men; <0.63, 0.63-1.03, ≥1.03 for women). Multiple logistic regression models were used to examine the association of these obesity indicators with IFG, after adjusting for age and other potential confounding factors, including gender, lifestyle (smoking status, alcohol intake, and exercise), HDL-C, family history of diabetes and HOMA-IR. Odds ratios (ORs) and 95% confidence intervals (CIs) were calculated. Data was analyzed using SAS version 9.0 (SAS Inc., Cary, NC, USA). All statistical tests were two-sided and statistical significance was determined as p<0.05.

## RESULTS

A total of 27,549 subjects (17,180 men and 10,369 women) were determined eligible for this study. The mean age was 43.2 years for men and 42.6 years for women. The mean WC was 84.1 cm for men and 73.8 cm for women while the mean BMI was 24.0 kg/m^2^ for men and 22.4 kg/m^2^ for women.

General characteristics of tertile groups of adiponectin are presented in Table 1. All variables showed significant differences among tertile groups of adiponectin (p<0.001) except age and exercise in men and smoking and exercise in women (Table 1).

In the highest tertile groups of WC and BMI, age-adjusted prevalence rates of IFG were higher in men than women (18.3% and 18.6% in men, 11.1% and 11.0% in women). In the lowest tertile group of adiponectin, age-adjusted prevalence rates of IFG were similar to those in the highest tertile groups of WC and BMI (18.8% in men, 11.8% in women) (Tables 2, 3).

After adjusting for age, adiponectin, hypertension, HDL-C, family history of diabetes and lifestyle, the ORs (95% CI) for IFG in the highest tertiles of WC and BMI compared to those in the lowest tertile in men were 1.83 (1.57 to 2.15) and 1.13 (0.97 to 1.32), respectively. After adjusting for age, WC, BMI, hypertension, HDL-C, family history of diabetes and lifestyle, the OR (95% CI) for IFG in the lowest tertile of adiponectin compared to the highest tertile was 1.87 (1.68 to 2.08) (Table 2, Model 1).

The corresponding ORs (95% CIs) of WC, BMI, and adiponectin in women were 1.60 (1.17 to 2.19), 1.30 (0.96 to 1.75), and 2.37 (1.93 to 2.91), respectively (Table 3, Model 1). After further adjustment for the HOMA-IR, the OR (95% CI) for IFG in the highest tertile of WC compared to the lowest tertile was 1.31 (1.11 to 1.54) and 1.25 (0.90 to 1.73) in men and women. The OR (95% CI) for IFG in the lowest tertile of adiponectin compared to the highest tertile was 1.76 (1.58 to 1.97) and 2.17 (1.76 to 2.68) in men and women (Tables 2, 3, Model 2). After further adjustment for the HOMA-IR, the association of WC with IFG disappeared in women and weakened, but was still significant, in men. However, associations between adiponectin and IFG remained significant even after further adjustment for the HOMA-IR in both men and women (Tables 2, 3, Model 2). BMI was negatively associated with IFG in men (Table 2, Model 2).

**Figure 1:**
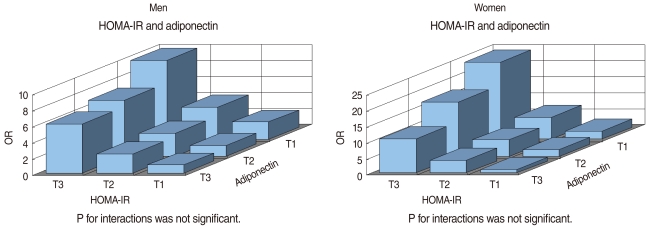
Combined association of insulin resistance (IR) assessed by the homeostasis model assessment of insulin resistance (HOMA-IR) and adiponectin levels with impaired fasting glucose (IFG) in men and women, expressed as multivariable adjusted odds ratios (ORs).

Strong combined effects of the IR and adiponectin with IFG were observed in both men and women. Multivariate adjusted ORs (95% CI) among those in the highest tertile of the HOMA-IR and the lowest tertile of adiponectin were 9.8 (7.96 to 12.07) and 24.1 (13.86 to 41.94) in men and women, when compared to those in the lowest tertile of the HOMA-IR and the highest tertile of adiponectin, respectively (Figure 1).

## DISCUSSION

Our findings showed that adiponectin and IR were strongly associated with IFG in a non-diabetic Korean population. These associations were independent of age, smoking status, alcohol consumption, exercise, HDL-C, Hypertension, family history of diabetes, WC, and BMI. Therefore, these results suggest that adiponectin may be a useful biomarker for diagnosing IFG.

In the present study, for those in the lowest tertile group of WC, the age-adjusted prevalence rates of IFG were substantially high at 9.0% for men and 7.2% for women. Although WC is highly correlated with abdominal obesity, those in the lowest tertile of WC are hardly considered to be in the high risk group for IFG. Similar phenomena such as high-risk individuals being 'metabolically obese but normal weight (MONW)' have been previously reported [19]. Hence, the risk of IFG might differ according to adiponectin levels in subjects with normal WC.

Indeed, it has been reported that adiponectin is closely related to insulin sensitivity, independent of fat mass [16]. A possible role for decreased adiponectin in the pathogenesis of IR was provided in a study by Hotta et al. [20] in Rhesus monkeys, which reported that plasma levels of adiponectin decreased in parallel with the development of IR. Adiponectin also reverses IR in animal models of obesity and diabetes [21]. In humans, hypoadiponectinemia has been associated with IR [22] and longitudinally with an increased risk of type 2 diabetes [23]. The glucose-lowering effect of adiponectin has been shown to be due in part to its activation of the AMP-activated protein kinase (AMPK) cascade. AMPK, a likely target for metformin and other antidiabetic drugs as well as for exercise-related glucose transport, is an insulin independent, phylogenetically ancient mechanism of stimulating glucose transport [23].

In the present study, both adiponectin and IR were independently associated with IFG. If IR was a mediator between adiponectin and IFG, then the association between adiponectin and IFG should be attenuated when adjusted for the HOMA-IR. However, in the present study, the association between adiponectin and IFG was not attenuated. The results reflect the fact that both adiponectin and IR may independently contribute to the development of IFG in this study population.

Neither WC nor BMI was substantially associated with IFG when adiponectin was included in the logistic models (Tables 2 and 3). In other words, adiponectin was the only significant predictor for IFG, and WC and BMI were not. Perhaps paradoxically, there is some evidence to suggest that omental fat may be a more important determinant of circulating adiponectin levels than subcutaneous abdominal fat, as adiponectin is secreted from human omental (but not subcutaneous) adipocytes [24]. Therefore, based on the results of the present study, the omental fat is more likely associated with adiponectin levels than either WC or BMI.

In recent studies, a low plasma adiponectin concentration was reported as a sensitive predictor of IFG in the development of diabetes [25] and high adiponectin levels were associated with a reduced risk of developing diabetes [26,27]. A survey found adiponectin provided extra-predictive power beyond obesity while leptin did not independently predict the risk of diabetes and IFG in older Chinese adults [28]. In the Hoorn study, a high adiponectin level was strongly associated with a lower risk of impaired glucose metabolism and type 2 diabetes, particularly in women [29]. However, only a few studies have investigated the association between adiponectin and IFG in those with normal WC. The present study also further confirmed that there were significant combined associations of adiponectin and the HOMA-IR with IFG (Figure 1), showing a robust association among those in the lowest tertile of adiponectin and highest tertile of HOMA-IR. Therefore, adiponectin can be considered as an additional marker to detect IFG, especially taken together with the HOMA-IR to predict the future onset of diabetes. Of course, our results need to be confirmed in future studies using cohort groups with IFG.

There are several limitations associated with WC measurements in this study. A high percentage of abdominal fat detected by computed tomography scan may present as normal WC, therefore WC is not a good indicator of abdominal fat. Also, due to the cross-sectional study design, it is impossible to assert a cause and effect relationship with regards to serum adiponectin and IR with IFG.

In conclusion, these results suggest that together, adiponectin and the HOMA-IR were strongly associated with IFG, supporting adiponectin as an additional marker for diagnosis of IFG.

## Figures and Tables

**Table 1:**
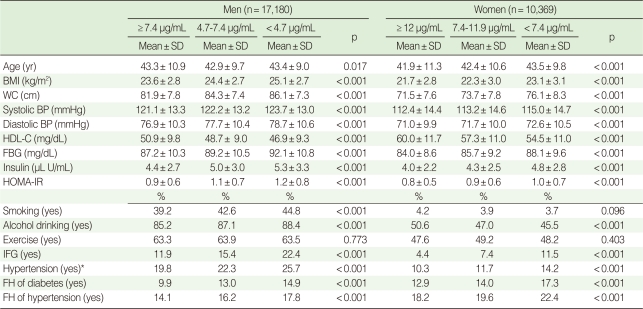
General study population characteristics according to adiponectin level

SD, standard deviation; BMI, body mass index; WC, waist circumference; BP, blood pressure; HDL-C, high density lipoprotein cholesterol; FBG, fasting blood glucose; HOMA-IR, homeostasis model assessment of insulin resistance; IFG, impaired fasting glucose; FH, family history. ^*^Hypertension=systolic blood pressure ≥140 mmHg, or diastolic blood pressure ≥90 mmHg, or on medication.

**Table 2:**
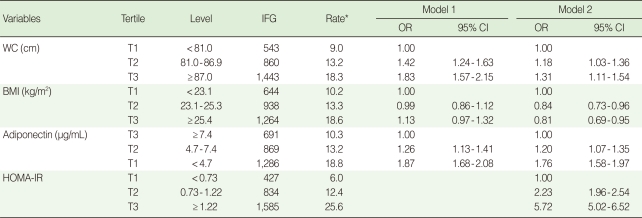
Multivariable adjusted odds ratios (ORs) and 95% confidence intervals (CIs) for IFG by tertile of WC, BMI, and adiponectin in men

6.52Model 1=Adjusted for age, WC, BMI, adiponectin, hypertension, smoking status, alcohol intake, exercise, HDL-C and FH of diabetes; Model 2=Model 1+HOMA-IR. IFG, impaired fasting glucose; WC, waist circumference; BMI, body mass index; HOMA-IR, homeostasis model assessment of insulin resistance. ^*^Age adjusted prevalence (%) of IFG.

**Table 3:**
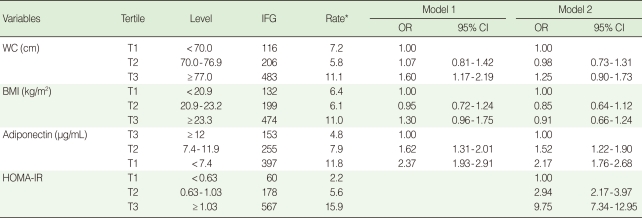
Multivariable adjusted odds ratios (ORs) and 95% confidence intervals (CIs) for IFG by tertile of WC, BMI, and adiponectin in women

Model 1=Adjusted for age, WC, BMI, adiponectin, hypertension, smoking status, alcohol intake, exercise, HDL-C and FH of diabetes; Model 2=Model 1+HOMA-IR. IFG, impaired fasting glucose; WC, waist circumference; BMI, body mass index; HOMA-IR, homeostasis model assessment of insulin resistance. ^*^Age adjusted prevalence (%) of IFG.
